# Prevalence and Antimicrobial Resistance of Pathogens Associated with Aerobic Vaginitis: A 10-Year Study in Greece

**DOI:** 10.3390/jcm15134926

**Published:** 2026-06-25

**Authors:** Anthia Chasiakou, Stamatia Chasiakou, George Kaparos, Vasiliki-Georgia Prifti, Stiliani Demeridou, Athanasios Tsakris, Stavroula Baka

**Affiliations:** 1 Department of Biopathology, Aretaieion University Hospital, National and Kapodistrian University of Athens, 76 Vassilisis Sofias Avenue, 11528 Athens, Greece; schasiakou@gmail.com (S.C.); captenkap@yahoo.gr (G.K.); vayiaprifti@yahoo.com (V.-G.P.); demeridou@yahoo.gr (S.D.); 2 Department of Microbiology, Medical School, National and Kapodistrian University of Athens, 75 Mikras Asias Street, 11527 Athens, Greece; atsakris@med.uoa.gr

**Keywords:** aerobic vaginitis, pathogens, antimicrobial resistance, I-dONE system

## Abstract

**Background**: Aerobic vaginitis (AV) is characterized by dysbiotic vaginal microflora with overgrowth of aerobic pathogens of enteric origin, presence of vaginal inflammation and immature epithelial cells. This study aimed to evaluate, over a period of 10 years, women of reproductive age (non-pregnant and pregnant) as well as menopausal women affected by AV. **Methods**: We included non-pregnant, pregnant and menopausal women diagnosed with AV over a period of 10 years. Diagnosis of AV was determined according to the criteria proposed by Donders in 2002. The isolated pathogens were identified with the rapid identification system I-dOne (Alifax S.r.l, Polverara, Italy) and the automated system VITEK2 (Biomerieux, Marcy l’Etoile, France), which was used for antimicrobial susceptibility testing. **Results**: The overall aerobic vaginitis prevalence rate during the studied period was 9.5%. The most common isolated pathogens were *Escherichia coli* 27.3%, *Enterococcus faecalis* 25.0%, *Streptococcus agalactiae* 22.2%, *Klebsiella pneumoniae* 8.9%, *Proteus* spp 4.7%, and *Staphylococcus aureus* 3.5%. *E. coli* infection significantly increased the odds of mild AV by 1.65 times (*p* = 0.002) and *Proteus* species infection was over 6 times more likely to progress to severe disease (*p* < 0.001). Furthermore, pregnant women were more likely to be infected with *E. faecalis* (*p* < 0.001) while menopausal women were diagnosed significantly more with severe AV (*p* < 0.001) compared to the other groups. **Conclusions**: The prevalence of aerobic vaginitis in the population studied was in concordance with global rates. Menopausal women displayed increased severe AV cases while, in contrast, mild cases were recorded during pregnancy. The most commonly isolated pathogens were of enteric origin.

## 1. Introduction

The vaginal milieu is a complex microenvironment with significant implications on women’s health. Any imbalance in the vaginal microbiome with significant decrease in lactobacilli species can result in genital tract infections, and, if left untreated, probably in infertility or decreased fertilization rates [1] or complications during pregnancy [2,3].

Aerobic vaginitis (AV) was first described in 2002 as a novel clinical entity in order to cover a different condition different from the ones already described [4]. It is characterized by dysbiotic vaginal microflora with overgrowth of aerobic pathogens of enteric origin, presence of vaginal inflammation and immature epithelial cells [5]. Although a non-life-threatening condition, it can affect quality of life in women of all ages and increase the susceptibility to sexually transmitted infections. Furthermore, there is a significant association with pelvic inflammatory disease as well as with adverse pregnancy outcomes (chorioamnionitis, miscarriage, preterm birth) [5,6]. It affects 5–30.7% of non-pregnant women [7,8,9,10] and 4.2–24.1% of pregnant women [11,12,13,14]. AV remains nowadays an underdiagnosed clinical entity and, even after more than two decades after its original description, very little data can be found in the literature. However, in 2018 the World Health Organization addressed the importance of AV by including guidelines regarding appropriate diagnosis and treatment which were updated in 2023 [15,16].

AV diagnosis is based on wet-mount phase-contrast microscopy through a specific scoring system that also grades the severity of AV [4]. Moreover, vaginal culture adds to diagnosis by pinpointing the exact responsible pathogen leading to an efficient therapeutic management after performing the antimicrobial susceptibility testing.

This study aimed to evaluate, over a period of 10 years, women of reproductive age (non-pregnant and pregnant) as well as menopausal women affected by AV, focusing on the isolated pathogens and the associated antimicrobial resistance.

## 2. Materials and Methods

This study was designed as an observational analysis of all pathogens isolated from vaginal cultures of women presenting with signs and symptoms of vaginitis and diagnosed with aerobic vaginitis by clinical and laboratory findings over a period of 10 years, 2016 to 2025. We included women of reproductive age, 18 years or older, non-pregnant and pregnant, as well as women at menopause, who were divided into the respective 3 groups. Data from all subjects were anonymous before analysis. Our study was based on the laboratory management data and was not directly associated with patients and, as a result, informed consent was not required as well as approval from our institution’s Ethics Committee.

Vaginal specimens were collected from all subjects using three sterile cotton-tipped swabs for wet-mount smear, Gram-stained smear and culture. The wet-mount smear was prepared by immediately placing the first swab in a tube containing 0.5 mL normal saline, and an appropriate amount of the suspension was then placed on a slide, covered with a coverslip and examined under a phase-contrast microscope at 400× magnification. The Gram-stained smear was prepared by rolling the second swab on a slide, fixing by flame, staining according to the manufacturer’s instructions (BioGnost, Zagreb, Croatia), and examining under microscope at both 400× and 1000× magnifications. The third swab, placed in Stuart’s bacterial transport medium, was used for culture. Wet-mount and Gram-stained smears helped to assess for the presence of *Trichomonas vaginalis*, blastospores, hyphae or pseudohyphae, leukocytes, epithelial cells, clue cells, and the microbial flora present in the vaginal microenvironment, in particular, the presence of *Lactobacillus* species, very important representatives of the normal vaginal flora that unfortunately decrease or even disappear in menopausal women. Cases diagnosed as trichomoniasis and vulvovaginal candidiasis were excluded from analysis.

Diagnosis of AV was primarily based on wet-mount phase-contrast microscopy. An AV score was determined according to lactobacillary grade, presence of leucocytes, toxic leucocytes, immature epithelial cells and vaginal microflora characteristics, as established by Donders et al. [4]. Lactobacillary grades were recorded as: grade I, numerous pleomorphic lactobacilli present and no other bacteria; IIa, mixed flora but predominantly lactobacilli; IIb, mixed flora and decreased lactobacilli; III, severely decreased or absent lactobacilli and overgrowth of other bacteria. The AV scoring system was used to differentiate between mild, moderate and severe AV since for each of these five characteristics, a score that ranged from 0 to 2 points was assigned and a composite score was obtained. A score of 0 to 2 was considered normal, 3 to 4 mild AV, 5 to 6 moderate AV, and a score > 6 corresponded to severe AV.

Bacterial vaginosis (BV) was diagnosed using Gram-stained vaginal smears observed with light microscopy under oil at a magnification of 1000×, according to the standardized morphological score analysis of Nugent et al. [17]. BV was identified at a score of ≥7, and these cases were excluded from this study.

Furthermore, vaginal samples were inoculated onto appropriate plates for standard aerobic and anaerobic cultures, which were incubated at 37 °C for 24 h and 48 h, respectively. All microorganisms classified as pathogens were grown as a single isolate or as the clear dominant bacteria on the culture plates. They were further identified based on colony morphology, color change of media around the colony, Gram staining, catalase test and bile-esculin test and confirmed with the rapid identification system I-dOne (Alifax S.r.l, Polverara, Italy) based on ATR-FTIR (Attenuated Total Reflection–Fourier Transform Infrared) spectroscopy. Finally, the automated system VITEK2 (Biomerieux, Marcy l’Etoile, France) was used for the definitive identification and antimicrobial susceptibility testing of all the isolated pathogens.

### Statistical Analysis

Statistical analysis was performed using SPSS version 22.0 (IBM Corp., Chicago, IL, USA). Descriptive statistics were used to summarize the data and are presented as frequencies and percentages. Cross-tabulation analyses were performed to examine associations between categorical variables. Odds ratios (ORs) with corresponding 95% confidence intervals (95% CIs) were calculated from 2 × 2 contingency tables using the Risk Estimate procedure in SPSS. Custom non-parametric binomial tests for proportions were applied where appropriate. A two-sided *p*-value < 0.05 was considered statistically significant.

## 3. Results

### 3.1. Prevalence of Aerobic Vaginitis

In this study, a total of 9216 vaginal samples were analyzed and 877 women were diagnosed with aerobic vaginitis. As a result, the overall prevalence rate of this clinical entity was 9.5%, with fluctuations over the 10-year period of this study ranging from 7.2 to 10.9% (Table 1).

### 3.2. Demographics and Clinical Characteristics of Patients

Out of the 877 women included in the study, 473 (53.9%) were of reproductive age and non-pregnant, 148 (16.9%) were pregnant and 256 (29.2%) were menopausal. The patients’ demographics and clinical characteristics are presented in Table 2. Most of the patients were of Greek origin (83.7%) and were married (74.9%). The most prevalent clinical symptom in all three groups was vaginal discharge, found in 97.1% of women, followed by vulvovaginal pruritus (52.9%) and vulvovaginal burning sensation (36.3%). In pregnant women, compared to the other groups, vulvovaginal pruritus was more frequent [OR = 1.749, 95% CI: (1.213–2.522), *p* = 0.003], while in contrast, vulvodynia [OR = 0.143, 95% CI: (0.045–0.459), *p* < 0.001], dyspareunia [OR = 0.274, 95% CI: (0.098–0.765), *p* = 0.008] and vulvovaginal burning sensation [OR = 0.488, 95% CI: (0.325–0.734), *p* < 0.001] were less frequently reported. Interestingly, menopausal women were 2.2 times more likely to report vulvovaginal burning sensation compared to the other groups [OR = 2.268, 95% CI: (1.683–3.058), *p* < 0.001].

### 3.3. AV Severity

Regarding the severity of disease, the majority of women (60.2%) displayed mild aerobic vaginitis. In the non-pregnant group we found no association with the level of disease severity. However, pregnant women displayed more mild AV cases [OR = 2.154, 95% CI: (1.448–3.203), *p* < 0.001], and fewer moderate [OR = 0.597, 95% CI: (0.397–0.899), *p* = 0.013] or severe cases [OR = 0.223, 95% CI: (0.069–0.719), *p* = 0.006] compared to the other groups. In contrast, in the menopausal group there were fewer mild cases [OR = 0.633, 95% CI: (0.471–0.850), *p* = 0.002] and more severe cases [OR = 2.915, 95% CI: (1.749–4.857), *p* < 0.001], when compared to the other groups.

### 3.4. Microorganisms Isolated

The isolated pathogens in the three study groups are described in Table 3. Among the isolated strains, 53.6% were Gram-positive cocci and 46.4% Gram-negative rods. The most common isolated microorganisms were *Escherichia coli* (27.3%), *Enterococcus faecalis* (25.0%), *Streptococcus agalactiae* (22.2%), *Klebsiella pneumoniae* (8.9%), *Proteus* species (4.7%) and *Staphylococcus aureus* (3.5%).

In the non-pregnant group no association was found with any of the isolated pathogens. Interestingly, patients in the pregnant group were more likely to be infected with *E. faecalis* [OR = 1.964, 95% CI: (1.347–2.863), *p* < 0.001], while on the contrary, menopausal patients had fewer *E. faecalis* infections [OR = 0.492, 95% CI: (0.339–0.715), *p* < 0.001], compared to the other groups. However, *E. faecalis* infection was not found to influence disease severity. Finally, menopausal women were also almost 2.5 times more likely to be infected by *Proteus* spp. [OR = 2.421, 95% CI: (1.289–4.549), *p* = 0.005]. When we compared the most frequently isolated pathogens with the severity of disease, *E. coli* infection increased the odds of mild disease by 1.65 times [OR = 1.646, 95% CI: (1.201–2.255), *p* = 0.002]. Also, patients with *E. coli* infection were less likely to progress to moderate disease [OR = 0.675, 95% CI: (0.485–0.980), *p* = 0.02]. On the other hand, *S. agalactiae* vaginitis was less associated with severe AV [OR = 0.401, 95% CI: (0.180–0.892), *p* = 0.021], while patients with *K. pneumoniae* infection were two times more likely to be diagnosed with moderate AV [OR = 2.017, 95% CI: (1.262–3.224), *p* = 0.003] and less likely with mild AV [OR = 0.505, 95% CI: (0.316–0.807), *p* = 0.004]. Notably, *Proteus* spp. infection was over six times more likely to cause severe AV [OR = 6.113, 95% CI: (2.952–12.660), *p* < 0.001] and two times more likely to cause moderate AV [OR = 2.063, 95% CI: (1.100–3.872), *p* = 0.022], although less likely to cause mild AV [OR = 0.172, 95% CI: (0.081–0.365), *p* < 0.001].

### 3.5. Antimicrobial Susceptibility

The resistance rates of the most commonly isolated pathogens, *E. coli*, *E. faecalis* and *S. agalactiae*, are presented in Table 4, Table 5 and Table 6, respectively. *E. coli* isolates were universally susceptible to imipenem, meropenem, colistin and tigecycline. Only five strains over the 10-year period were resistant to ertapenem. Among aminoglycosides, amikacin showed lower resistance rates and the same was true for piperacillin-tazobactam. Increased and almost-constant resistance rates to fluoroquinolones were found over the studied period, reaching up to 40%. In contrast, ampicillin was the least active antimicrobial against *E. coli*, while an increasing resistance trend was noted for cephalosporins (Table 4).

As expected, among Gram-positive cocci, both *E. faecalis* and *S. agalactiae* were universally susceptible to vancomycin, teicoplanin and linezolid. Furthermore, no resistance to penicillin and tigecycline was found among *S. agalactiae* strains. Very few *E. faecalis* isolates were resistant to ampicillin, while increasing rates were recorded for erythromycin and fluoroquinolones (Table 5). On the other hand, tetracycline demonstrated very low susceptibility rates against *S. agalactiae* isolates, while erythromycin and clindamycin displayed increasing resistance rates over the years. Specifically, clindamycin resistance varied from 5.9 to 22.6%, and erythromycin from 11.7 to 33.3% (Table 6).

## 4. Discussion

Vaginal flora has a significant and beneficial effect on women’s health. Any alterations of the vaginal milieu can facilitate infections. For decades, the established vaginal infections were bacterial vaginosis, vulvovaginal candidiasis and trichomoniasis. In 2002, aerobic vaginitis was included as another imbalance in the vaginal ecosystem resulting in infection due to aerobic pathogens [4]. In Europe, AV prevalence ranges between 5 and 12% [5,18]. Our results are in concordance with these data and with previous reports from Greece [19,20], although increased prevalence has been reported in non-pregnant (23.8%) and pregnant (24.1%) women, as well [13]. However, it is important to point out that the prevalence rates were affected by the outbreak of the COVID-19 pandemic. We documented decreased number of samples during 2020 and 2021 due to restricted access to healthcare services. During lockdowns, although medical visits were permitted, social distancing guidelines and contagion fears led to a decline in outpatient visits.

AV diagnosis is mainly based on specific criteria proposed by Donders et al. [4]. Nevertheless, a positive vaginal culture can prove helpful for diagnosis and adequate treatment options [16]. It cannot be overlooked that the pathogens isolated in AV cases are commonly found in the vaginal flora of healthy women. However, women diagnosed with AV are symptomatic and aerobic pathogens are grown as the single or dominant microorganism from their vaginal cultures. A common finding in previous reports and in our study was that the most frequently isolated pathogens were *E. coli*, *K. pneumoniae* and *Proteus* spp., among Gram-negative rods, and *E. faecalis*, *S. agalactiae* and *S. aureus* among Gram-positive cocci [13,18,19,20,21]. In our study population, the most prevalent symptoms were vaginal discharge, vulvovaginal pruritus and burning sensation as previously described [4,5].

We included women of reproductive age, both pregnant and non-pregnant, as well as women at menopause. Almost one-third of the population included in this study comprised menopausal women. Menopause induces several changes in the vaginal microenvironment increasing the incidence of vaginitis and even severe AV cases, as documented in this study. Increased prevalence in women at menopause could be explained by the hormonal imbalance and decrease in lactobacilli, findings commonly encountered with increasing age [5]. In our study, a marked predominance of *Pseudomonas aeruginosa* isolates among menopausal women was recorded. *P. aeruginosa* is not a microorganism usually encountered in a healthy vaginal microbiome, nor a typical pathogen associated with vaginitis. However, in menopausal women, decreased estrogen levels induce vaginal atrophy, elevate vaginal pH, and compromise *Lactobacillus* dominance through the reduction or depletion of glycogen reserves, allowing the overgrowth of virulent pathogens not typically associated with vaginitis, such as *P. aeruginosa* [1,2,5,20].

Data regarding AV severity are scarce in the literature. It has been estimated that the prevalence of moderate and severe AV is around 7–13% in non-pregnant women [5,22,23]. Contrary to our results, Rumyantseva et al. found lower prevalence rates of mild (13.4%), moderate (7.2%) and severe (3.1%) AV cases in non-pregnant women [24]. An interesting finding in our patients was the impact that *Proteus* spp. had on the severity of disease. Although the observation was performed on a relatively low number of isolates, *Proteus* spp. infection was over six times more likely to cause severe AV. *Proteus* spp., usually considered common commensals of the gastrointestinal microbiota, are also opportunistic pathogens responsible for many infections. Their many virulence factors, including adhesins, swarming motility, production of urease and proteases, as well as the ability of biofilm formation, are essential for the pathogenesis of *Proteus* infections [25]. In particular, for vaginal infections, a crucial role can be attributed to the urease enzyme that metabolizes urea into ammonia, a rich source of nitrogen for microbial metabolism and survival. Ammonia increases vaginal pH, thus allowing the survival of bacteria in a more acidic environment. Additionally, ammonia is extremely cytotoxic to vaginal cells, promoting substantial tissue damage and progression to severe infections [25]. The presence of moderate and severe AV has been linked to sexually transmitted diseases and to major Pap smear abnormalities [5], significantly increasing when high-risk human papilloma virus genotypes were present [23]. Moreover, AV has been associated with pelvic inflammatory disease and infertility [26]. As a result, this clinical entity must be included in the differential diagnosis algorithm of vaginal infections. On the other hand, the significance of AV during pregnancy regarding the complications that can occur has been addressed; hence, moderate and severe AV represents an increased risk factor for adverse pregnancy outcomes, such as spontaneous miscarriage, preterm pre-labor rupture of membranes, chorioamnionitis and preterm delivery [11,14,26,27].

Undoubtedly, antimicrobials have proved over decades to be one of the most important tools to treat many bacterial infections. Antimicrobial resistance is not a new phenomenon and has been acknowledged as a global health burden. As a result, continuous surveillance of antimicrobial resistance is imperative to guide therapeutic strategies in patients. Apart from colistin and tigecycline, which are not first-line antimicrobials, carbapenems and aminoglycosides were the most effective against *E. coli* isolates, consistent with previous reports [13,21,28]. The increased resistance to cephalosporins and fluoroquinolones is a matter of concern that has been already pointed out [21,29]. As previously reported from Greece [13] and Italy [21], in this study all *E. faecalis* isolates were susceptible to vancomycin, teicoplanin, and linezolid, and displayed high susceptibility rates to ampicillin. However, the increased resistance rates seen in fluoroquinolones mirrors their increased consumption practiced in Greece over the last decades [19]. Our study has documented that all *S. agalactiae* strains were susceptible to penicillin, which is the first-line antimicrobial recommended for this pathogen. In patients allergic to penicillin, clindamycin, erythromycin, fluoroquinolones and vancomycin are recommended [30]. In line with previous data [13], we experienced increasing resistance rates to erythromycin and clindamycin. This phenomenon is alarming on a global level and has been addressed after their widespread use [31,32]. Fluoroquinolone resistance was a rare event in our study population. In contrast, tetracycline remained broadly resistant, which explains why this antimicrobial is no longer recommended [31,33,34]. Finally, we confirm that vancomycin retained its excellent activity on *S. agalactiae* strains.

This study has several limitations that should be acknowledged. First, it must be taken into account that the presented data was obtained from one tertiary, university-based hospital; hence, the results may be interpreted to a certain extent. Second, the antimicrobial treatment administered and clinical outcomes were unavailable; thus, the long-term consequences, such as the recurrence rates of vaginitis, were not evaluated.

## 5. Conclusions

This work aimed to add to the limited data available from our country regarding AV prevalence in different groups of women, the isolated pathogens and their resistance patterns. AV proved to be a measurable cause of vaginal infections that should always be taken into consideration for differential diagnosis. Much attention is necessary to infections occurring during pregnancy and in women of childbearing age in general, in order to protect and prevent any possible transmission to the fetus and/or any further adverse outcome on the reproductive tract, if any. Therefore, in order to control antimicrobial resistance, it is important to monitor, through epidemiological studies, the pathogens most commonly isolated in AV cases, as well as their susceptibility trends, in order to apply the optimal therapeutic choices in each specific patient. Future multicenter studies involving large cohorts and longer follow-up post-treatment periods are warranted to document a more targeted therapeutic approach and the impact of AV on vaginal health.

## Figures and Tables

**Table 1:** Vaginal samples analyzed during the study period.

Year	Total Samples	Positive Samples
2016	1242	135 (10.9%)
2017	1224	119 (9.7%)
2018	968	98 (10.1%)
2019	874	68 (7.8%)
2020	635	46 (7.2%)
2021	670	71 (10.6%)
2022	889	93 (10.5%)
2023	947	90 (9.5%)
2024	923	85 (9.2%)
2025	844	72 (8.5%)
Total	9216	877 (9.5%)

**Table 2:** Demographics and clinical characteristics of AV patients included in the study. Numbers are *n* (%).

Variables	Reproductive Age and Non-Pregnant	Pregnant	Menopausal
Number of patients, 877 (100)	473 (53.9)	148 (16.9)	256 (29.2)
Age (years, mean)	34.75	34.36	61.39
Nationality			
Greeks, 734 (83.7)	392 (82.9)	113 (76.4)	229 (89.5)
Immigrants, 143 (16.3)	81 (17.1)	35 (23.6)	27 (10.5)
Marital status			
Married, 657 (74.9)	289 (61.1)	146 (98.6)	222 (86.7)
Unmarried, 220 (25.1)	184 (38.9)	2 (1.4)	34 (13.3)
Pregnancy trimester			
First	-	45 (30.4)	-
Second	-	55 (37.2)	-
Third	-	48 (32.4)	-
Clinical symptoms			
Vaginal discharge, 852 (97.1)	461 (97.5)	148 (100)	243 (94.9)
Vulvovaginal pruritus, 464 (52.9)	237 (50.1)	95 (64.2)	132 (51.6)
Vulvovaginal burning sensation, 318 (36.3)	155 (32.8)	35 (23.6)	128 (50.0)
Vulvodynia, 95 (10.8)	56 (11.8)	3 (2.0)	36 (14.1)
Dyspareunia, 71 (8.1)	40 (8.5)	4 (2.7)	27 (10.5)
AV severity			
Mild, 528 (60.2)	284 (60.0)	110 (74.3)	134 (52.3)
Moderate, 284 (32.4)	161 (34.0)	35 (23.6)	88 (34.4)
Severe, 65 (7.4)	28 (5.9)	3 (2.0)	34 (13.3)

Abbreviation: AV: aerobic vaginitis.

**Table 3:** Distribution of pathogens isolated in the study groups.

Pathogens	Reproductive Age and Non-Pregnant	Pregnant	Menopausal	Total
**Gram-positive cocci**				
*Staphylococcus aureus*	12	7	12	31 (3.5%)
*Staphylococcus lugdunensis*	4	0	1	5 (0.6%)
*Staphylococcus haemolyticus*	0	1	0	1 (0.1%)
*Enterococcus faecalis*	123	54	42	219 (25.0%)
*Enterococcus faecium*	7	1	6	14 (1.6%)
*Enterococcus avium*	1	0	0	1 (0.1%)
*Streptococcus agalactiae*	112	26	57	195 (22.2%)
*Streptococcus pyogenes*	2	0	0	2 (0.2%)
*Streptococcus sanguinis*	1	0	0	1 (0.1%)
*Streptococcus parasanguinis*	0	1	0	1 (0.1%)
*Streptococcus gallolyticus*	1	0	0	1 (0.1%)
**Gram-negative rods**				
*Escherichia coli*	129	38	72	239 (27.3%)
*Klebsiella pneumoniae*	48	13	17	78 (8.9%)
*Klebsiella oxytoca*	4	0	2	6 (0.7%)
*Klebsiella aerogenes*	5	0	2	7 (0.8%)
*Proteus* spp.	18	2	21	41 (4.7%)
*Enterobacter cloacae*	0	1	0	1 (0.1%)
*Pseudomonas aeruginosa*	1	1	16	18 (2.1%)
*Pseudomonas oleovorans*	1	0	0	1 (0.1%)
*Pseudomonas fluorescens*	0	0	1	1 (0.1%)
*Citrobacter freundii*	1	0	2	3 (0.3%)
*Citrobacter koseri*	1	2	1	4 (0.5%)
*Citrobacter braaki*	0	1	0	1 (0.1%)
*Stenotrophomonas maltophilia*	0	0	1	1 (0.1%)
*Morganella morganii*	1	0	3	4 (0.5%)
*Serratia marcescens*	0	0	1	1 (0.1%)

**Table 4:** Antimicrobial resistance patterns of *E. coli* isolates. Numbers are *n* (%).

Antimicrobial	2016	2017	2018	2019	2020	2021	2022	2023	2024	2025
Ampicillin	12 (63.2)	12 (66.7)	14 (63.7)	18 (72.0)	17 (70.8)	17 (65.4)	23 (82.1)	23 (85.2)	20 (87.0)	24 (88.9)
Amoxicillin- clavulanate	6 (31.6)	7 (38.9)	8 (36.4)	10 (40.0)	9 (37.5)	8 (30.8)	12 (42.9)	11 (40.7)	10 (43.5)	10 (37.0)
Piperacillin- tazobactam	2 (10.5)	1 (5.6)	2 (9.1)	2 (8.0)	2 (8.3)	3 (11.5)	3 (10.7)	4 (14.8)	2 (8.7)	3 (11.1)
Cefotaxime	4 (21.1)	4 (22.2)	4 (18.2)	7 (28.0)	8 (33.3)	11 (42.3)	13 (46.4)	11 (40.7)	11 (47.8)	12 (44.4)
Ceftazidime	2 (10.5)	3 (16.7)	3 (13.6)	5 (20.0)	5 (20.8)	7 (26.9)	9 (32.1)	9 (33.3)	7 (30.4)	8 (29.6)
Cefepime	2 (10.5)	2 (11.1)	2 (9.1)	2 (8.0)	3 (12.5)	4 (15.4)	6 (21.4)	6 (22.2)	6 (26.1)	7 (25.9)
Ertapenem	0	0	0	1 (4.0)	0	1 (3.8)	1 (3.6)	0	1 (4.3)	1 (3.7)
Imipenem	0	0	0	0	0	0	0	0	0	0
Meropenem	0	0	0	0	0	0	0	0	0	0
Gentamicin	3 (15.8)	3 (16.7)	5 (22.7)	5 (20.0)	3 (12.5)	5 (19.2)	5 (17.9)	4 (14.8)	3 (13.0)	5 (18.5)
Tobramycin	3 (15.8)	2 (11.1)	5 (22.7)	4 (16.0)	2 (8.3)	4 (15.4)	4 (14.3)	3 (11.1)	3 (13.0)	4 (14.8)
Amikacin	1 (5.2)	1 (5.5)	1 (4.5)	1 (4.0)	1 (4.2)	1 (3.8)	1 (3.6)	1 (3.7)	1 (4.3)	1 (3.7)
Ciprofloxacin	7 (36.8)	7 (38.9)	9 (40.9)	9 (36.0)	9 (37.5)	10 (38.5)	9 (32.1)	9 (33.3)	9 (39.1)	9 (33.3)
Levofloxacin	5 (26.3)	5 (27.8)	5 (22.7)	5 (20.0)	6 (25.0)	7 (26.9)	6 (21.4)	5 (18.5)	4 (17.4)	7 (25.9)
Ofloxacin	7 (36.8)	7 (38.9)	9 (40.9)	9 (36.0)	9 (37.5)	10 (38.5)	9 (32.1)	9 (33.3)	9 (39.1)	9 (33.3)
Tigecycline	0	0	0	0	0	0	0	0	0	0
Colistin	0	0	0	0	0	0	0	0	0	0
Trimethoprim- sulfamethoxazole	3 (15.8)	3 (16.7)	4 (18.2)	5 (20.0)	4 (16.7)	5 (19.2)	6 (21.4)	8 (29.6)	7 (30.4)	9 (33.3)

**Table 5:** Antimicrobial resistance patterns of *E. faecalis* isolates. Numbers are *n* (%).

Antimicrobial	2016	2017	2018	2019	2020	2021	2022	2023	2024	2025
Ampicillin	0	0	1 (4.5)	1 (5.0)	1 (3.7)	1 (4.8)	1 (5.5)	1 (4.5)	1 (3.6)	1 (3.7)
Vancomycin	0	0	0	0	0	0	0	0	0	0
Teicoplanin	0	0	0	0	0	0	0	0	0	0
Erythromycin	12 (63.2)	12 (80.0)	17 (77.3)	18 (90.0)	25 (92.6)	20 (95.2)	18 (100)	21 (95.5)	28 (100)	27 (100)
Tetracycline	5 (26.3)	4 (26.7)	5 (22.7)	5 (25.0)	7 (25.9)	5 (23.8)	5 (27.8)	5 (22.7)	7 (25.0)	6 (22.2)
Ciprofloxacin	4 (21.1)	4 (26.7)	4 (18.2)	6 (30.0)	9 (33.3)	9 (42.9)	7 (38.9)	10 (45.5)	15 (53.6)	15 (55.6)
Levofloxacin	3 (15.8)	3 (20.0)	5 (22.7)	6 (30.0)	9 (33.3)	10 (47.6)	9 (50.0)	11 (50.0)	13 (46.4)	14 (51.9)
Chloramphenicol	0	0	0	0	0	0	0	0	0	0
Linezolid	0	0	0	0	0	0	0	0	0	0
Ampicillin-sulbactam	0	0	0	0	0	0	0	0	0	0

**Table 6:** Antimicrobial resistance patterns of *S. agalactiae* isolates. Numbers are *n* (%).

Antimicrobial	2016	2017	2018	2019	2020	2021	2022	2023	2024	2025
Penicillin	0	0	0	0	0	0	0	0	0	0
Erythromycin	2 (14.3)	2 (16.7)	2 (11.7)	5 (23.8)	3 (20.0)	6 (25.0)	6 (33.3)	5 (31.3)	8 (29.6)	10 (32.3)
Clindamycin	1 (7.1)	1 (8.3)	1 (5.9)	2 (9.5)	2 (13.3)	5 (20.8)	4 (22.2)	3 (18.8)	6 (22.2)	7 (22.6)
Levofloxacin	0	0	0	0	0	1 (4.2)	0	0	1 (3.7)	0
Tetracycline	11 (78.6)	10 (83.3)	13 (76.5)	17 (80.9)	14 (93.3)	21 (87.5)	16 (88.9)	15 (93.8)	25 (92.6)	28 (90.3)
Vancomycin	0	0	0	0	0	0	0	0	0	0
Linezolid	0	0	0	0	0	0	0	0	0	0
Teicoplanin	0	0	0	0	0	0	0	0	0	0
Tigecycline	0	0	0	0	0	0	0	0	0	0

## Data Availability

All data generated or analyzed during this study are available from the corresponding authors on reasonable request.

## Abbreviations

The following abbreviations are used in this manuscript:

AV	Aerobic vaginitis
BV	Bacterial vaginosis
ATR-FTIR	Attenuated Total Reflection–Fourier Transform Infrared

## References

[1] Saraf V.S., Sheikh S.A., Ahmad A., Gillevet P.M., Bokhari H., Javed S. (2021). Vaginal microbiome: Normalcy vs dysbiosis. Arch. Microbiol..

[2] Chopra C., Bhushan I., Mehta M., Koushal T., Gupta A., Sharma S., Kumar M., Al Khodor S., Sharma S. (2022). Vaginal microbiome: Considerations for reproductive health. Future Microbiol..

[3] Ncib K., Bahia W., Leban N., Mahdi A., Trifa F., Mzoughi R., Haddad A., Jabeur C., Donders G. (2022). Microbial diversity and pathogenic properties of microbiota associated with aerobic vaginitis in women with recurrent pregnancy loss. Diagnostics.

[4] Donders G.G.G., Vereecken A., Bosmans E., Dekeersmaecker A., Salembier G., Spitz B. (2002). Definition of a type of abnormal vaginal flora that is distinct from bacterial vaginosis: Aerobic vaginitis. BJOG Int. J. Obstet. Gynaecol..

[5] Donders G.G., Bellen G., Grinceviciene S., Ruban K., Vieira-Baptista P. (2017). Aerobic vaginitis: No longer a stranger. Res. Microbiol..

[6] Rezeberga D., Lazdane G., Kroica J., Sokolova L., Donders G.G. (2008). Placental histological inflammation and reproductive tract infections in a low risk pregnant population in Latvia. Acta Obstet. Gynecol. Scand..

[7] Fan A., Yue Y., Geng N., Zhang H., Wang Y., Xue F. (2013). Aerobic vaginitis and mixed infections: Comparison of clinical and laboratory findings. Arch. Gynecol. Obstet..

[8] Marconi C., Donders G.G.G., Bellen G., Brown D.R., Parada C.M.G.L., Silva M.G. (2013). Sialidase activity in aerobic vaginitis is equal to levels during bacterial vaginosis. Eur. J. Obstet. Gynecol. Reprod. Biol..

[9] Aklilu A., Woldemariam M., Manilal A., Koira G., Alahmadi R.M., Raman G., Idhayadhulla A., Yihune M. (2024). Aerobic vaginitis, bacterial vaginosis, and vaginal candidiasis among women of reproductive age in Arba Minch, southern Ethiopia. Sci. Rep..

[10] Mishra G., Gupta K., Mohanty S., Mitra S., Jena S. (2025). Evaluation of various diagnostic modalities for detection of bacterial vaginosis and aerobic vaginitis: An underdiagnosed entity among women of the reproductive age group. Indian J. Pathol. Microbiol..

[11] Donders G.G., Van Calsteren K., Bellen G., Reybrouck R., Van den Bosch T., Riphagen I., Van Lierde S. (2009). Predictive value for preterm birth of abnormal vaginal flora, bacterial vaginosis and aerobic vaginitis during the first trimester of pregnancy. Br. J. Obstet. Gynecol..

[12] Zodzika J., Rezeberga D., Jermanova I., Vasina O., Vedmedovska N., Donders G. (2011). Factors related to elevated vaginal pH in the first trimester of pregnancy. Acta Obstet. Gynecol. Scand..

[13] Tansarli G.S., Skalidis T., Legakis N.J., Falagas M.E. (2017). Abnormal vaginal flora in symptomatic non-pregnant and pregnant women in a Greek hospital: A prospective study. Eur. J. Clin. Microbiol. Infect. Dis..

[14] Han C., Li H., Han L., Wang C., Yan Y., Qi W., Fan A., Wang Y., Xue F. (2019). Aerobic vaginitis in late pregnancy and outcomes of pregnancy. Eur. J. Clin. Microbiol. Infect. Dis..

[15] Sherrard J., Wilson J., Donders G., Mendling W., Jensen J.S. (2018). 2018 European (IUSTI/WHO) International Union against sexually transmitted infections (IUSTI) World Health Organisation (WHO) guideline on the management of vaginal discharge. Int. J. STD AIDS.

[16] Sherrard J., Wilson J., Donders G., Mendling W., Jensen J.S. (2023). 2023 update to 2018 European (IUSTI/WHO) guideline on the management of vaginal discharge. Int. J. STD AIDS.

[17] Nugent R.P., Krohn M.A., Hillier S.L. (1991). Reliability of diagnosing bacterial vaginosis is improved by a standardized method of gram stain interpretation. J. Clin. Microbiol..

[18] Tansarli G.S., Kostaras E.K., Athanasiou S., Falagas M.E. (2013). Prevalence and treatment of aerobic vaginitis among non-pregnant women: Evaluation of the evidence for an underestimated clinical entity. Eur. J. Clin. Microbiol. Infect. Dis..

[19] Iavazzo C., Vogiatzi C., Falagas M. (2008). A retrospective analysis of isolates from patients with vaginitis in a private Greek obstetric/gynecological hospital (2003–2006). Med. Sci. Monit..

[20] Sianou A., Galyfos G., Moragianni D., Baka S. (2017). Prevalence of vaginitis in different groups among females in Greece. J. Obstet. Gynecol..

[21] Serretiello E., Santella B., Folliero V., Iervolino D., Santoro E., Manente R., Dell’Annunziata F., Sperlongano R., Crudele V., De Filippis A. (2021). Prevalence and antibiotic resistance profile of bacterial pathogens in aerobic vaginitis: A retrospective study in Italy. Antibiotics.

[22] Dermendjiev T., Pehlivanov B., Hadjieva K., Stanev S. (2015). Epidemiological, clinical and microbiological findings in women with aerobic vaginitis. Akush. Ginekol..

[23] Vieira-Baptista P., Lima-Silva J., Pinto C., Saldanha C., Beires J., Martinez-de-Oliveira J., Donders G. (2016). Bacterial vaginosis, aerobic vaginitis, vaginal inflammation and major Pap smear abnormalities. Eur. J. Clin. Microbiol. Infect. Dis..

[24] Rumyantseva T.A., Bellen G., Savochkina Y.A., Guschin A.E., Donders G.G. (2016). Diagnosis of aerobic vaginitis by quantitative real-time PCR. Arch. Gynecol. Obstet..

[25] Hasona I.F., Awad A., Younis G., Mohamed W.F. (2026). A One Health perspective on *Proteus mirabilis*: The interaction of virulence and antimicrobial resistance across human and animal reservoirs. Microorganisms.

[26] Han C., Wu W., Fan A., Wang Y., Zhang H., Chu Z., Wang C., Xue F. (2015). Diagnostic and therapeutic advancements for aerobic vaginitis. Arch. Gynecol. Obstet..

[27] Donders G.G.G., Bellen G., Rezeberga D. (2011). Aerobic vaginitis in pregnancy. BJOG Int. J. Obstet. Gynaecol..

[28] Foglia F., Della Rocca M.T., Montella F., Vasco M., Chianese A., Zannella C., De Filippis A., Finamore E., Galdiero M. (2024). Aerobic vaginitis: Antibiotic resistance trend and future actions of antimicrobial diagnostic stewardship. New Microbiol..

[29] Pullingam T., Parumasivam T., Gazzali A.M., Sulaiman A.M., Chee J.Y., Lakshmanan M., Chin C.F., Sudesh K. (2022). Antimicrobial resistance: Prevalence, economic burden, mechanisms of resistance and strategies to overcome. Eur. J. Pharm. Sci..

[30] The American College of Obstetricians and Gynecologists (2020). Prevention of group B streptococcal Early-Onset disease in newborns: ACOG committee opinion, number 797. Obstet. Gynecol..

[31] Sabroske E.M., Sanson Iglesias M.A., Rench M., Moore T., Harvey H., Edwards M., Baker C.J., Flores A.R. (2023). Evolving antibiotic resistance in group B streptococci causing invasive infant disease: 1970–2021. Pediatr. Res..

[32] Hsu C.Y., Moradkasani S., Suliman M., Uthirapathy S., Zwamel A.H., Hjazi A., Vashishth R., Beig M. (2025). Global patterns of antibiotic resistance in group B *Streptococcus*: A systemic review and meta-analysis. Front. Microbiol..

[33] Hayes K., O’Halloran F., Cotter L. (2020). A review of antibiotic resistance in Group B *Streptococcus*: The story so far. Crit. Rev. Microbiol..

[34] Kaminska D., Ratajczak M., Nowak-Malczewska D.M., Karolak J.A., Kwasniewski M., Szumala-Kakol A., Dlugaszewska J., Gajecka M. (2024). Macrolide and lincosamide resistance of *Streptococcus agalactiae* in pregnant women in Poland. Sci. Rep..

